# The Undecided Have the Key: Interaction-Driven Opinion Dynamics in a Three State Model

**DOI:** 10.1371/journal.pone.0139572

**Published:** 2015-10-05

**Authors:** Pablo Balenzuela, Juan Pablo Pinasco, Viktoriya Semeshenko

**Affiliations:** 1 Departamento de Física, Facultad de Ciencias Exactas y Naturales, Universidad de Buenos Aires and IFIBA, CONICET, Buenos Aires, Argentina; 2 Departamento de Matemática, Facultad de Ciencias Exactas y Naturales, Universidad de Buenos Aires and IMAS UBA-CONICET, Buenos Aires, Argentina; 3 Consejo Nacional de Investigaciones Científicas y Técnicas (CONICET), Buenos Aires, Argentina; 4 Instituto Interdisciplinario de Economía Política de Buenos Aires (IIEP-BAIRES), Facultad de Ciencias Económicas, Universidad de Buenos Aires, Buenos Aires, Argentina; Uppsala University, SWEDEN

## Abstract

The effects of interpersonal interactions on individual’s agreements result in a social aggregation process which is reflected in the formation of collective states, as for instance, groups of individuals with a similar opinion about a given issue. This field, which has been a longstanding concern of sociologists and psychologists, has been extended into an area of experimental social psychology, and even has attracted the attention of physicists and mathematicians. In this article, we present a novel model of opinion formation in which agents may either have a strict preference for a choice, or be undecided. The opinion shift emerges, in a threshold process, as a consequence of a cumulative persuasion for either one of the two opinions in repeated interactions. There are two main ingredients which play key roles in determining the steady states: the initial fraction of undecided agents and the change in agents’ persuasion after each interaction. As a function of these two parameters, the model presents a wide range of solutions, among which there are consensus of each opinion and bi-polarization. We found that a minimum fraction of undecided agents is not crucial for reaching consensus only, but also to determine a dominant opinion in a polarized situation. In order to gain a deeper comprehension of the dynamics, we also present the theoretical framework of the model. The master equations are of special interest for their nontrivial properties and difficulties in being solved analytically.

## Introduction

When a group of inter-related individuals discuss around a given item, they are prone to change their initial opinions in order to get similar to or dissimilar from other subjects in the group. This interpersonal dynamics leads to different consequences which can be categorised either by consensus or coexistence of opinions. Furthermore, if the topic is a binary statement, as for example a pro-against issue, the coexistence of opinions turns out to be a polarized state, where a fraction of agents holds a given opinion and the rest of the group takes the opposite one.

These kind of situations naturally lead to questions like: What are the mechanisms leading to the formation of these collective states? Can we predict the final collective outcomes when different mechanisms compete among each other, as for instance, when some individuals tend to agree and others to disagree?

A numerical modelling approach could be a powerful tool for facing these kind of questions and testing how an elected interaction mechanism between pair of agents leads to the formation of collective states. The typical approach to model the formation of opinion is to assume that an individual either adopts the opinion of a neighbour or decides based on an average of neighbouring opinions. We take a different conjecture, that the process of opinion formation emerges from an underlying dynamics. Here, we develop a novel model based on the combination of the interaction-based persuasion and a threshold-driven opinion change dynamics.

In sociological research, the concept of threshold was introduced in the seminal papers of T. Schelling [1] and M. Granovetter [2] when explaining the micro-macro link and the aggregation processes. From the psychological perspective, accumulative-threshold models have been successfully used [3, 4] in the analysis of binary decision making problems.

The formation of opinion’s collective states and mechanisms that generate them have been largely studied in sociology and social psychology [5–9] among others. There are five main theories that explain group opinion dynamics [10, 11]: Social comparison theory [5, 12, 13]; Information or Persuasive arguments theory (PAT) [14, 15]; Self-categorization theory [16–18]; Social decision theory [19–21] and Social influence network theory [10]. Mostly, these theories are focused on interpersonal interactions, and are useful because they draw attention to the emergence of communication patterns. Essentially, this deepens the understanding of the group dynamics, in particular, when combined with an appropriate mathematical model.

From a theoretical point of view, much of the existing modelling progress on opinion dynamics has been addressed within a physics-based framework, where the behavioural mechanism of social influence is derived from analogies with physical systems, in particular spins [22, 23]. The variety of existing models assume that individuals hold binary or continuous opinion values (usually distributed between -1 and 1), which are updated through repeated interactions between neighbouring agents. Different models assume different rules for opinion adaptation, such as imitation [8], averaging over individuals with similar opinions [24], the majority rule [25], or more sophisticated rules [26, 27]. The classical models [28–30] predict consensus, meanwhile models with mechanism of negative influence (disliking of dissimilar ones) naturally will give place to bi-polarization. However, in last years new models with interesting features appeared, as for instance, a model of continuous opinion based on persuasive arguments theory where bi-polarization can be produced without including negative influence explicitly [31]. Also, competition between the two antagonist mechanisms, as persuasion and compromise among agents with different degree of agreement about a given issue, has been explored in [32].

In this work we present an agent-based model for a population of interdependent individuals who simultaneously participate in an artificial interaction process. The individuals can have either one of the two opposing views, and also be undecided. After random encounters, agents may increase or decrease their persuasions depending on the opinion of the opponent [32, 33]. These social interactions produce cumulative changes that can eventually lead to the change of opinion: a shift in opinion occurs when the persuasion exceeds a certain threshold. Thus, the opinion formation is an emerging process which depends on the underlying dynamics of the persuasion of each individual in a given issue.

There are exist different models in the literature which consider the undecided individuals, see for instance ([34–37]). However, the model to be discussed in this paper has its novelty in the combined features mentioned above.

We analyse the equilibria reached by this population and the convergence properties of the system for a wide range of parameter’s values. Three main collective states can be seen: bi-polarization, consensus and convergence of undecided agents. Moreover, the model shows that the initial fraction of undecided agents could be crucial in the determination of consensus or the dominance of a given opinion in a polarized situation. We also analyse how the system can reach the mentioned collective states in a regime with initially low concentration of undecided individuals. Finally we sketch the exact dynamical equations in order to frame the model within the non-linear and non-local first order differential equation formulation.

The article is organized as follows: the model and the interaction dynamics are presented in section Analysis. The results are presented and discussed in section Results. We conclude in section Discussion and provide a theoretical development of master equations in Appendix.

## Analysis

In order to study the opinion formation process in groups we develop a simple agent-based model that includes the relevant features for modelling the opinion dynamics: positive social influence (in successive interactions individuals with the same opinion become more similar) and negative influence (disliking of dissimilar others).

We consider a population of *N* individuals (*i* = 1,2, …, *N*), each one simultaneously participating in interpersonal interactions, which can be understood as an exchange of some kind of information between two agents over the issue in question. The population has the following characteristics: each individual is represented as an agent *i*, and holds the persuasion *C*_*i*_. The variable *C*_*i*_ vary between *C*_*max*_ and −*C*_*max*_, and represents being fully aligned with one of the opinions or totally opposed with it.

In addition, agent *i* has an opinion *O*_*i*_ which stands for agent’s posture on a given issue at period *t*. The opinion variable *O*_*i*_ can take three values of attitude towards an issue: positive *O*_*i*_ = +1, negative *O*_*i*_ = −1, and neutral *O*_*i*_ = 0. Note that the negative opinion does not have a negative connotation about the issue in question. The negative sign is due to the numerical representation of the opinion.

**Fig 1:**
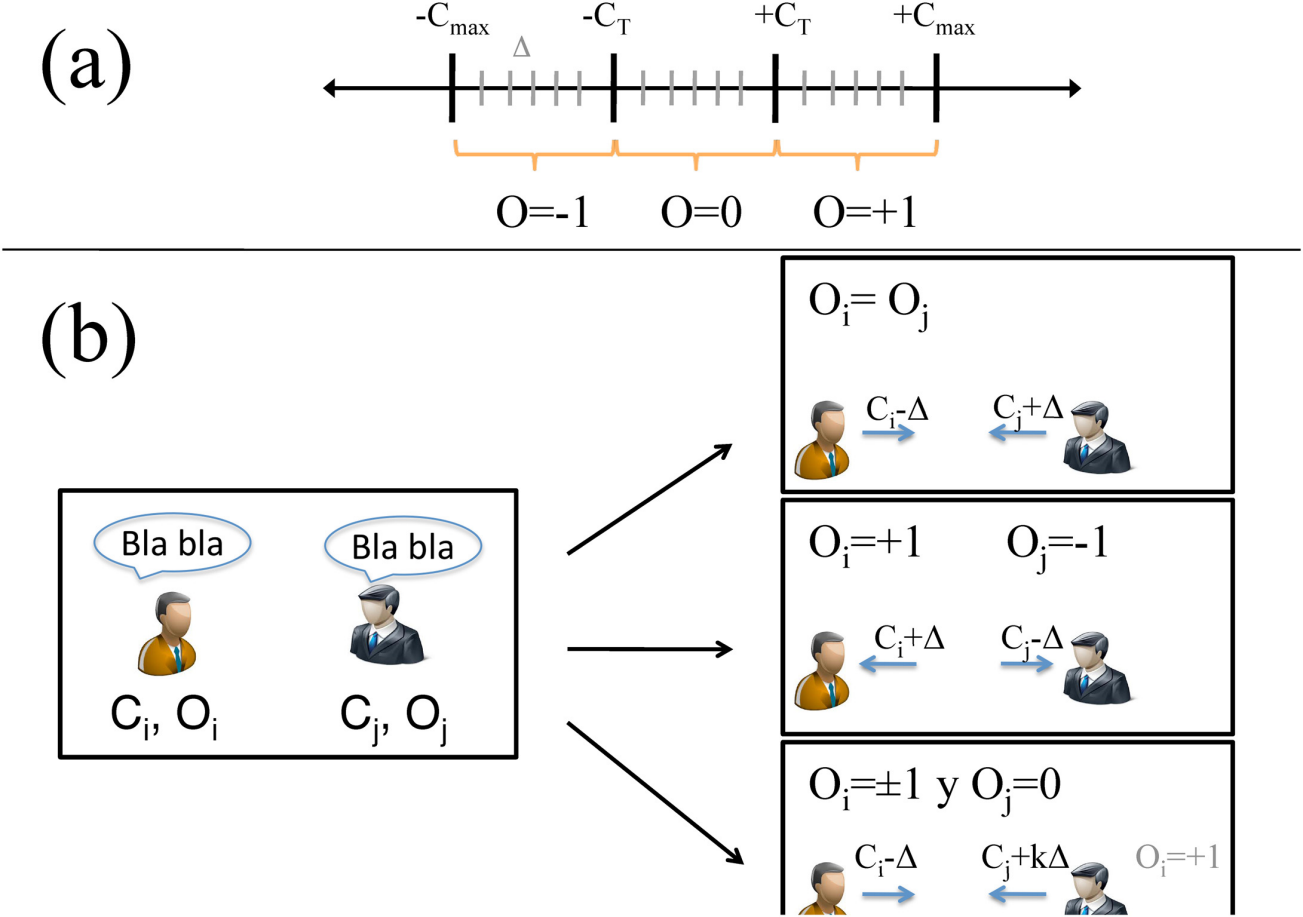
Schematic representation of the opinion dynamics. Panel (a): Relation between persuasion (*C*) and opinion (*O*). Each persuasion interval ([−*C*_*max*_, *C*_−*T*_], [−*C*_ − *T*_, *C*_*T*_],[*C*_*T*_, *C*_*max*_]) defines one of the three opinion values (*O* = −1, 0, +1 respectively). Moreover, each interval is divided in sub-intervals of length Δ. Panel (b): Description of how persuasion is modified via pairwise interactions in three different cases: Same opinion (top panel), opposing opinions (middle panel) and formed opinion vs undecided.

The opinion is fully determined by the persuasion of agent in the following way: If *C*_*i*_ is greater than a given positive threshold, *C*_*i*_ > *C*_*T*_, the agent has a positive opinion *O*_*i*_ = +1, and if it is less than a given negative threshold, *C*_*i*_ < −*C*_*T*_, the agent’s opinion is negative *O*_*i*_ = −1. If the agent is not convinced enough to decide with any definite position, then he is undecided and *O*_*i*_ = 0 (see Fig 1(a)).

### Dynamics

We are interested in the equilibria reached by the system when agents meet and interact in successive periods. We model this as a process where agents may increase or decrease their persuasions after each interaction depending on whether the opponent has positive or negative opinion. These social interactions produce cumulative changes that can eventually lead to the change of opinion: a shift in opinion occurs when the persuasion exceeds a given threshold.

The interaction dynamics between agents is the following: whenever two agents *i* and *j* interact, the agents’ persuasion values *C*_*i*_ and *C*_*j*_ are modified by an amount bounded by *k*Δ, depending on the opinion of both individuals. This parameter, Δ, is a measure of how an agent changes the persuasion after a given interaction.

This social influence mechanism works by moving the respective persuasions of their existing positions to new positions depending on the opponent. This way the opinion shift is not based on the imitation of opinions of the neighbours (like the typical imitation behaviours), but is due to a cumulative persuasive dynamics.

The way in which the two social ingredients of the interacting dynamics (positive and negative social influence) is implemented, will produce an “anti-flocking effect”: Dissimilar individuals repel (caused by mechanism of negative influence) if they differ in opinions and approach each other if their opinions are similar (positive social influence). This last is in line with the fact observed by Wood [38] where individuals sharing a common attribute tend to get closer in opinions.

More formally, at each time step *t*, the states of the agents after interaction are updated according to the following rules, also illustrated in Fig 1(b):

- If agents share the same opinions or are both undecided, *O*_*i*_(*t*) = *O*_*j*_(*t*), and *C*_*i*_(*t*) > *C*_*j*_(*t*) then they approach (agents influence each other so that their persuasions become closer)
  $$C_{i}\left(t+1\right)=C_{i}\left(t\right)-\Delta ,$$
  $$C_{j}\left(t+1\right)=C_{j}\left(t\right)+\Delta .$$
- If agents hold different opinions, *O*_*i*_(*t*) = +1, and *O*_*j*_(*t*) = −1 then they repel
  $$C_{i}\left(t+1\right)=C_{i}\left(t\right)+\Delta ,$$
  $$C_{j}\left(t+1\right)=C_{j}\left(t\right)-\Delta .$$
  If one of the agents has an opinion and another one is undecided, they approach. In this case the dynamics is asymmetric simulating the fact that is not the same convincing someone who does not have any opinion yet that making someone change his opinion.
- If *O*_*i*_ = ∓1 and *O*_*j*_ = 0 then
  $$C_{i}\left(t+1\right)=C_{i}\left(t\right)\pm \Delta ,$$
  $$C_{j}\left(t+1\right)=C_{j}\left(t\right)\mp k\Delta .$$

We also implement that if two agent *i* and *j* interacts and ∣*C*_*i*_ − *C*_*j*_∣ < Δ, then Δ = Δ_*eff*_, where $\Delta_{eff}=\frac{|C_{i}-C_{j}|}{2}$.

Summarising, the main parameters of the model are:

- Δ is a sensitivity parameter which measures how much the persuasion of an agent changes after each interaction. The smaller it is, the more interactions agent needs to get convinced.
- *P*_0_ is the initial fraction of undecided agents.
- *C*_*T*_ is the threshold beyond which the agent is no longer undecided and adopts an opinion.
- *k* is a variable which simulates an asymmetric dynamics between an undecided agent and the one with a formed opinion. Values *k* > 1 imply that the persuasion of the undecided is modified by a factor *k* with respect to the other one.

Given the details of the interaction dynamics, we are interested in the following question: Do the interactions among the agents with different opinions bring the group to consensus or bi-polarization?

We present the results of simulations in the next sections. All the simulations are done for systems with *N* = 10000 agents. Results are averages over ensembles *N*_*ev*_ = 100 equivalent configurations, corresponding to different realisations of the random initial conditions.

## Results

In this section we present the steady states of the model and discuss their properties as a function of the relevant parameters. The aggregate behaviour is characterised in terms of the fraction of agents who state an opinion *i*, *S*_*i*_, where *i* = {+, −, 0}. Given that *S*_*i*_(*t*) is a function of time, we call *P*_*i*_ ≡ *S*_*i*_(0) the initial fraction of agents who has an opinion *i*, and *T*_*i*_ ≡ *S*_*i*_(*t*_*asint*_) the fraction of agents who have come to a definite position on an issue or have “no opinion” at convergence.

There are two features of the model that, *a priori*, are preferred to be fixed: *first*, opinions should be equally likely; *second*, it is assumed to be easier for undecided agents to adopt an opinion because they get persuaded, than for those who have an opinion to change it and become “undecided”. The first feature is implemented in a way that once the initial fraction of undecided agents, *P*_0_, is chosen, the rest of the agents are equally distributed with either one of opinions (±1). When we change this condition, we will call *P*_+_ the fraction of agents with *O* = +1 after the undecided were assigned. The second feature is implemented by setting *k* = 2. The persuasions of agents are drawn at random from an uniform distribution within each opinion.

For better description and interpreting interaction effects is especially useful to take in Fig 1.

### Equilibrium States

We start with analysing the steady states *T*_*i*_ ≡ *S*_*i*_(*t*_*asint*_) as a function of *P*_0_ and Δ. We vary 0.01 ≤ *P*_0_ ≤ 0.99 and 0.01 ≤ Δ ≤ 1.0 with steps of 0.01. Equilibria *P*_0_ = 0 and *P*_0_ = 1 are already absorbing states, in both cases the interaction will not change the opinions of the agents. If initially all agents are undecided, they will remain undecided. On the contrary, if agents are equally distributed between the two extreme opinions, the distribution of opinions will remain unchanged.

Results are obtained with asynchronous updating where the procedure is iterated until convergence. The steady state is reached when *T*_0_ = 1 or *T*_0_ = 0, i.e. agents are either all undecided or all have an opinion.

As a result of simulations, the system converges to one of these three equilibrium states:

(a) *T*_0_: All undecided (*T*_0_ = 1).
(b) *T*_+_ *T*_−_: Consensus (either *T*_+_ = 1 or *T*_−_ = 1).
(c) *T*_*bp*_: Bi-Polarization (*T*_0_ = 0, 0 < *T*_+_ < 1 and 0 < *T*_−_ < 1).

Given the constraint *S*_0_(*t*) + *S*_+_(*t*) + *S*_−_(*t*) = 1 (∀*t*), if *T*_0_ = 0, the equilibria are either consensus of one of the extreme opinion (b) or bi-polarization (c).

**Fig 2:**
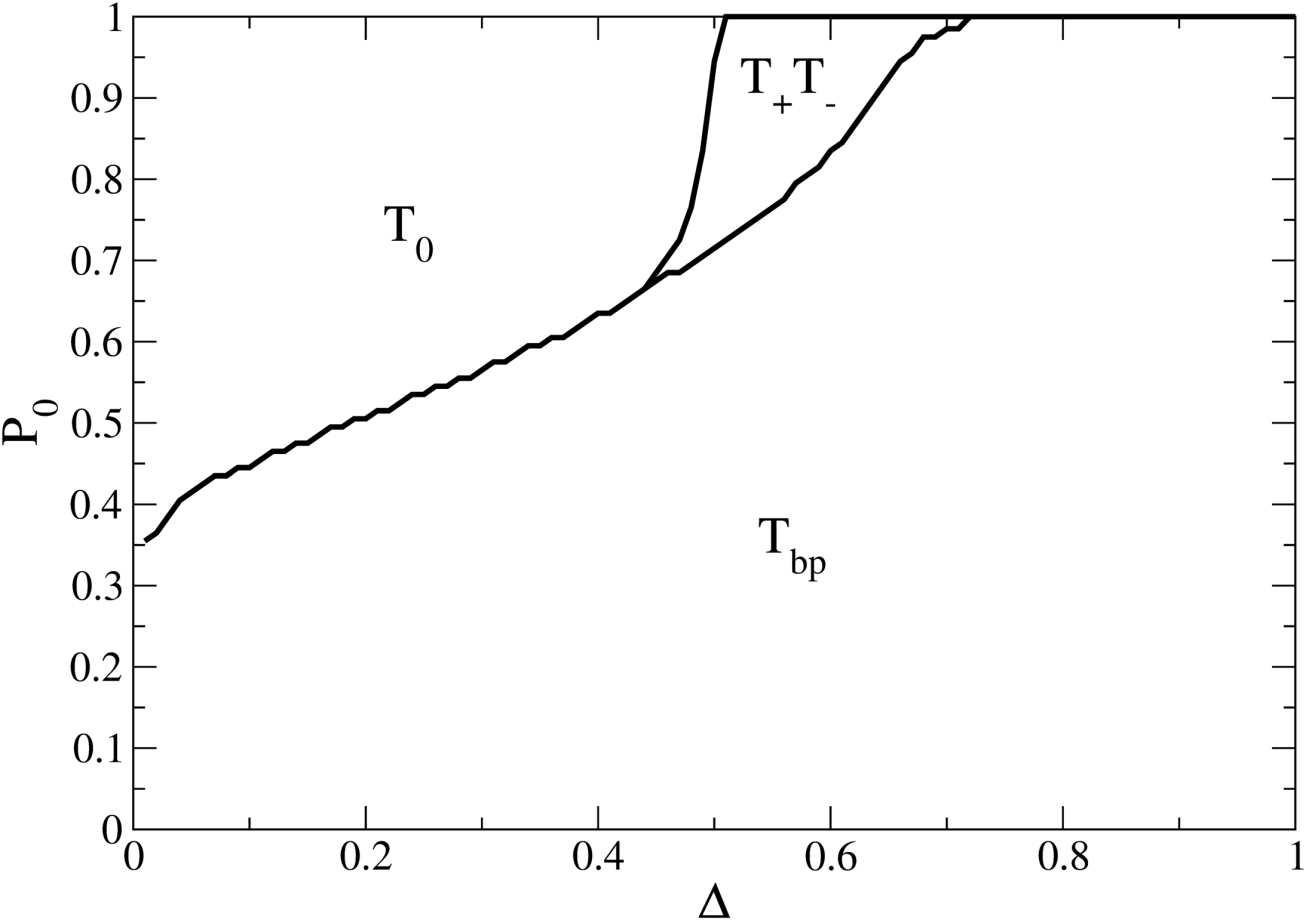
Fundamental Phase Diagram. Dominant steady state solution as a function of *P*_0_ and Δ. We identify three different regions: region *T*_0_, convergence of undecided; region *T*_+_ *T*_−_, consensus of opinions +1/-1 and region *T*_*bp*_, bi-polarization. In the simulations *C*_*T*_ = 1, *k* = 2, *C*_*max*_ = 3 were used.

Fig 2 shows the Fundamental Phase Diagram (*FPD*) that depicts existence of different regions of the system under equilibrium. In this Phase Diagram we analyse the prevalence of each equilibria for every pair of values of *P*_0_ and Δ within the specific range.

The Phase Diagram exhibits three different regions (see Fig 2):

*T*_0_, delimited by large values of *P*_0_ and small values of Δ, where the equilibrium state is characterised by convergence of undecided agents ( 〈*T*_0_〉 = 1).
*T*_+_ *T*_−_, delimited by large values of *P*_0_ and intermediate values of Δ, where the equilibrium state is characterised by consensus of either of the two opinions ( 〈*T*_+_〉 = 1 or 〈*T*_−_〉 = 1).
*T*_*bp*_, delimited by low values of *P*_0_ when Δ is small and by all values of *P*_0_ when Δ is large, where the steady state is defined by a bi-polarization of opinion ( 〈*T*_0_〉 = 0, 0 < 〈*T*_+_〉 < 1 and 0 < 〈*T*_−_〉 < 1).

The Fundamental Phase Diagram (*FPD*) can be understood in terms of the positive and negative social influence described before: interactions between agents sharing a common attribute (i.e., same opinion) force them get closer in persuasion *C* [38], bringing them to the center, meanwhile interactions between dissimilar agents, move them away from *T*_0_. The result of these two competing opposing forces depends on the initial fraction of undecided agents *P*_0_, and the sensitivity parameter Δ (see Fig 2).

When Δ is small, the situation is dominated by agents that change persuasions very little after pairwise interactions. Thus, many of these interactions are needed in order to produce a change in their opinions. Here, the fraction *P*_0_ is important because it determines what will be the final state of this dynamics (all undecided ( 〈*T*_0_〉 = 1) or none ( 〈*T*_0_〉 = 0)).

Instead, when Δ increases, the preference for an agent to adopt extreme opinions (due to the asymmetry given by *k*, see Fig 1(b)) is more evident and is reflected in the fact that the more initially undecided agents are needed in order for the system to reach the final state with “all undecided”. As a consequence, the border of region *T*_0_ grows monotonically with Δ until it encloses with *P*_0_ = 1. When Δ is large, the final state is bi-polarization (region *T*_*bp*_) independently of the initial density of undecided agents.

### Consensus and Bi-Polarization

Given the global picture of the stable solutions, we look into the details at the dynamics which makes the systems reach these states. Furthermore, we restrict the discussion to regions *T*_+_ *T*_−_ and *T*_*bp*_ where the equilibrium states are consensus and bi-polarization, respectively, because region *T*_0_ does not represent any interest neither from the social nor the individual point of view. For example, the statistics on undecided voters indicate that most individuals have pre-existing beliefs when it comes to politics, and relatively few ones remain undecided late into high-profile elections [39].

Let’s start with region *T*_*bp*_, which deserves a closer look. Phase Diagram (see Fig 2) only gives information about this region regarding to whether is dominated by the coexistence of opposing opinions, but it does not clarify if one opinion prevails, or both exist in equal parts.

**Fig 3:**
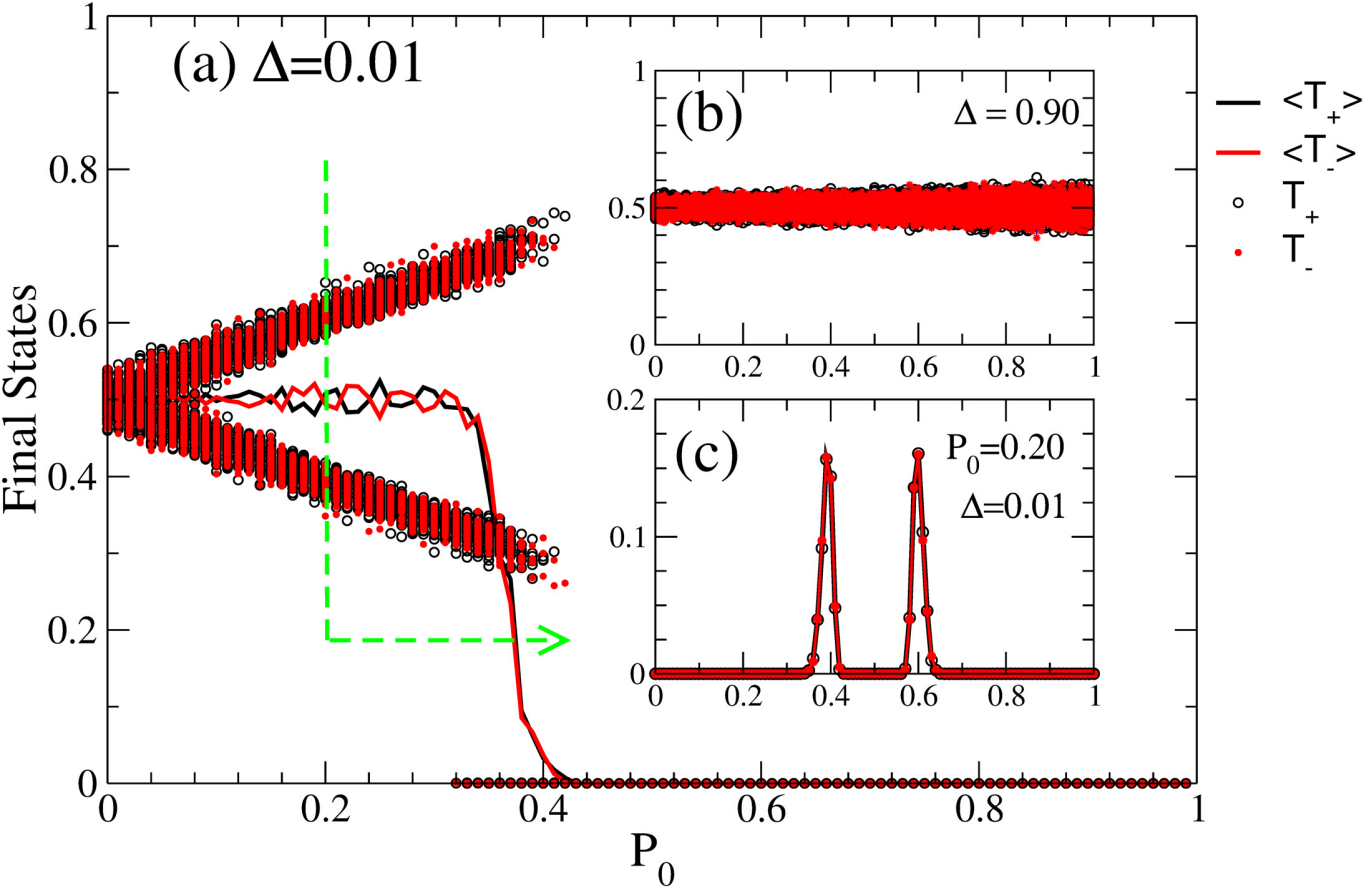
Analysis of Bi-polarization Region *T*_*bp*_. Main Panel (a): Distribution of positive (*T*_+_, black circles) and negative opinions (*T*_−_, red circles) and their averages ( 〈*T*_+_〉, 〈*T*_−_〉 black and red thick lines respectively) as a function of *P*_0_ for Δ = 0.01. The two branches in the bi-polarization regions mean the presence of dominant and dominated opinions for this value of Δ. Insets: Panel (b): Same quantities for Δ = 0.9. For large values of Δ, there does not exist a dominant opinion. Panel (c): Bimodal distribution of positive and negative final opinions for *P*_0_ = 0.20 and Δ = 0.01.

Fig 3 helps clarify these questions by showing that:

- For small values of Δ, both opinions exist, among which one is dominant and the other one is dominated. The degree of dominance depends on *P*_0_. This can be seen in the diverging branches (Fig 3, black and red circles), which represent *T*_+_ and *T*_−_ for different simulations at different values of *P*_0_ and Δ = 0.01. The distance between the branches follows a linear relation with *P*_0_, meaning that in the final state, the undecided agents go to one or the other opinion with the same probability, given that initially they were equally distributed.
- For large values of Δ, the distribution of opinions is about 50% each, as it can be seen in Fig 3, panel (b).
- Given that initially neither of the two opinions is preferred, both are equally likely. This is reflected in the fact that, on average, either of 〈*T*_+_〉 and 〈*T*_−_〉 are 50% (Fig 3, solid lines in main panel), but their distributions are bimodal. An example of this bimodal distribution can be observed in Fig 3, panel (c) for *P*_0_ = 0.20.

**Fig 4:**
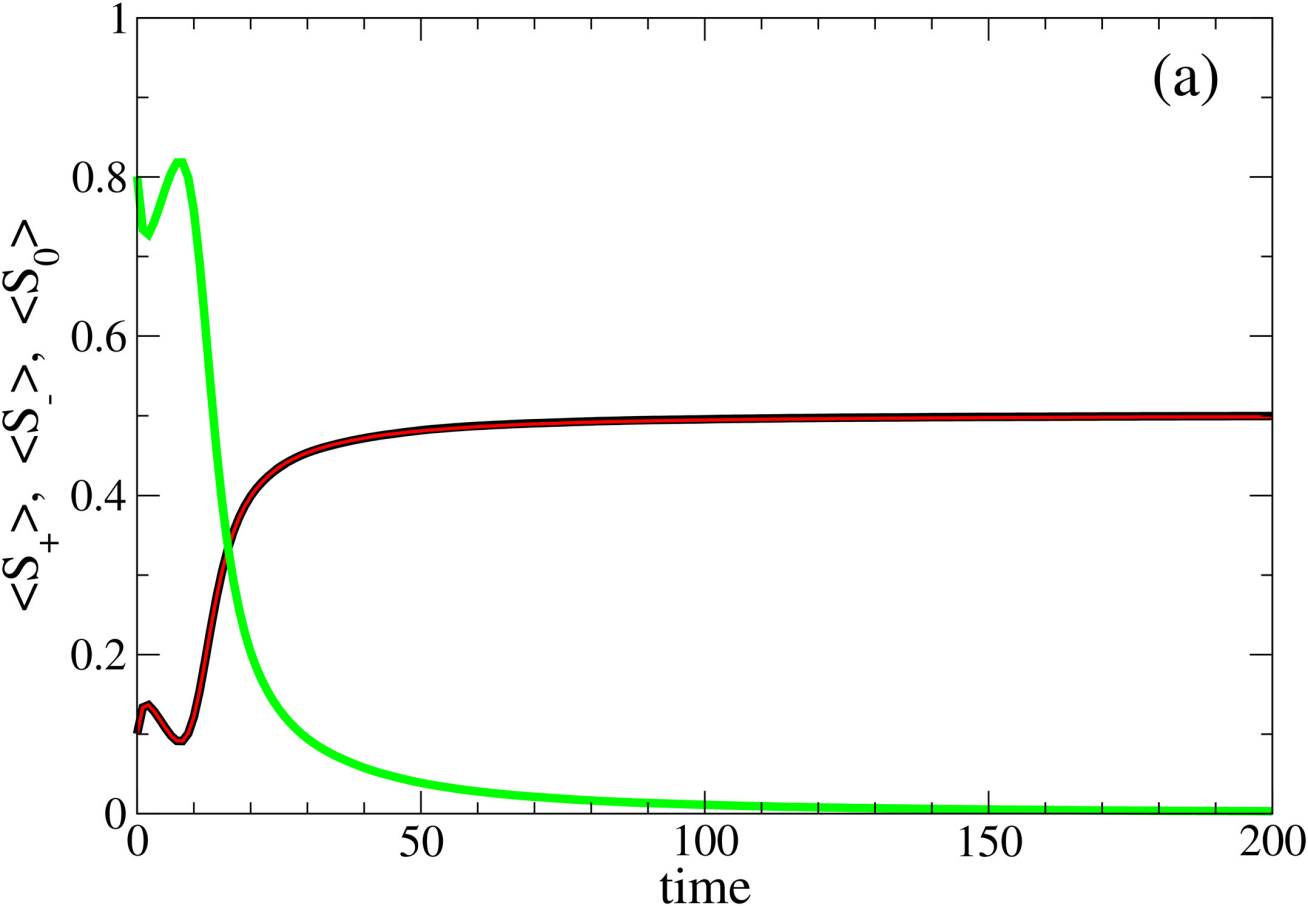
Analysis of Consensus Region (*T*_+_ *T*_−_). Averaged opinion dynamics for a representative point in region *T*_+_ *T*_−_ of the Fundamental Phase Diagram (*P*_0_ = 0.80, Δ = 0.55). The plot shows the averaged evolution in time for each opinion dynamics (< *S*_+_ >, black, < *S*_−_ >, red and < *S*_0_ >, green). It can be observed that undecided population grows until it reaches a value above 80% in the first time steps but finally one of the populations with defined opinions becomes dominant with the same probability. Evolutions are averaged over *N*_*ev*_ = 10000.

As was mentioned earlier, there are two competing mechanisms that act as persuasion forces and drive agents either towards the center (*C* = 0) or the extremes (*C* = ±*C*_*max*_), giving place to the two symmetric solutions: all undecided (*T*_0_) and bi-polarization (*T*_*bp*_). However, there exist a region of space parameter, *T*_+_ *T*_−_, where all agents adopt, equally likely, either one of the two extreme opinions, involving a symmetry breaking in the dynamics. This kind of behaviour takes place for high values of *P*_0_ and for intermediate values of Δ. In Fig 4, we plot the average fraction of agents with each opinion as a function of time, for a representative point of this region (*P*_0_ = 0.80 and Δ = 0.55).

In all the events the fraction of undecided agents, *S*_0_, goes to zero after an initial transient time. On the contrary, the fraction of agents with opinion, *S*_+_ and *S*_−_, prevails with the same probability and therefore their averages, 〈*S*_+_〉 and 〈*S*_−_〉, go to 0.5 (see Fig 4 black and red lines respectively).

**Fig 5:**
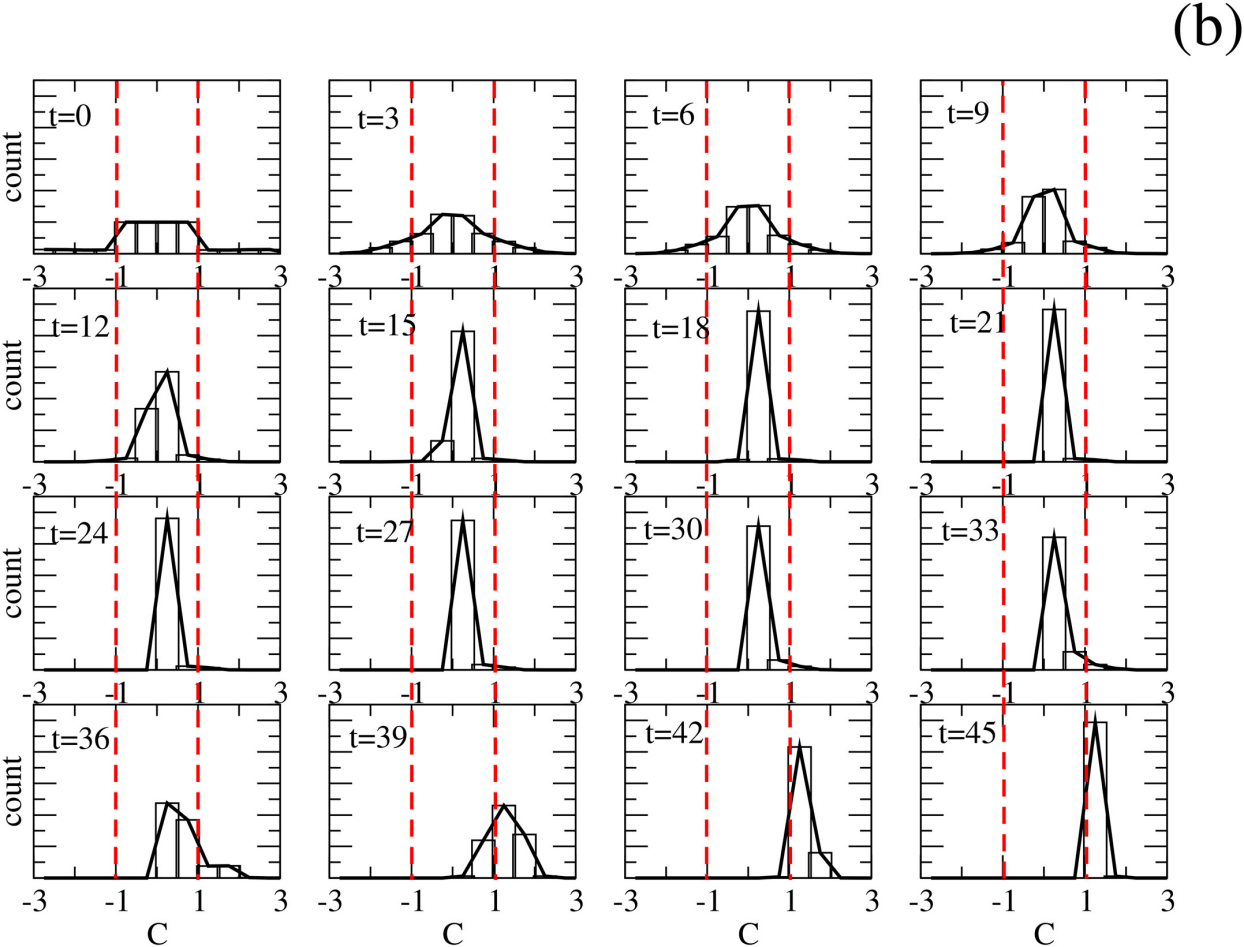
Analysis of Consensus Region (*T*_+_ *T*_−_). Evolution of Persuasion’s histograms for a single event. They show how undecided seems to win for times between 20 and 30, but the asymmetry in the persuasion distribution together with the mechanism described in the text makes all the agents adopt one of the opinions.

This behaviour can be understood if we retain that the change in agent’s opinion emerges from the interchanging dynamics of the persuasion variable *C*. In Fig 5, we plot the histograms of persuasion *C*, for different periods of the time dynamics of a single event, where all the agents reach consensus with opinion *O* = +1.

In order to make clear that the final dominant opinion does not appear from any little difference in initial conditions, we choose, for this single event, a perfectly symmetrical initial condition: 8000 undecided agents (half with *C* ≥ 0 and the other half with the same values of *C* but with opposite sign), 1000 agents with *O* = +1 and 1000 with *O* = −1 and the same (but negative) values of persuasion variable *C*. The distribution of *C* within the interval corresponding to each opinion is constant, as can be seen in the histogram for *t* = 0. We can see that, after perfectly symmetrical initial conditions, some undecided agents with persuasion value close to threshold adopt ±1 opinions but then a vast majority of agents became undecided. This can be understood because, given the high proportion of undecided, these drive the agents toward the center (near *C* = 0). However these new distributions are not perfectly symmetrical, as can be appreciated from times *t* = 9 to *t* = 27. At this point of the evolution the dynamics is driven by interactions between decided (*O* = +1 in this case) and undecided agents near the threshold *C*_*T*_. Here, we should remind that, when these types of agents interact, the decided agent changes its persuasion *C* by Δ while the undecided changes *C* by 2Δ. This asymmetry in interactions makes an initially undecided agent near the threshold to become “more decided” than an initially decided one near the threshold and the consequence after several interactions will be a drift from undecided to decided agents, producing the consensus in the population.

Summarising, the key of consensus in this model comes from this kind of interactions where an almost persuaded (but still undecided) agent interacts with a weakly decided one and, after the interaction, both adopt the same opinion but the initially undecided becomes more persuaded than the other one. This kind of mechanism describes situations where a new integrant of a group (in this case, those with *O* = +1) tries to act as a more authentic member of the group than genuine members of same group.

In order to analyse the role of the parameters on the solution of the model, we also explored the impact produced on the Phase Diagram when the threshold *C*_*T*_ and the asymmetry parameter *k* are modified for Δ = 0.01. For this value of Δ, the systems presents only two solutions: convergence of undecided agents or bi-polarization. Changing these two parameters does not modify qualitatively the solutions of the model.

**Fig 6:**
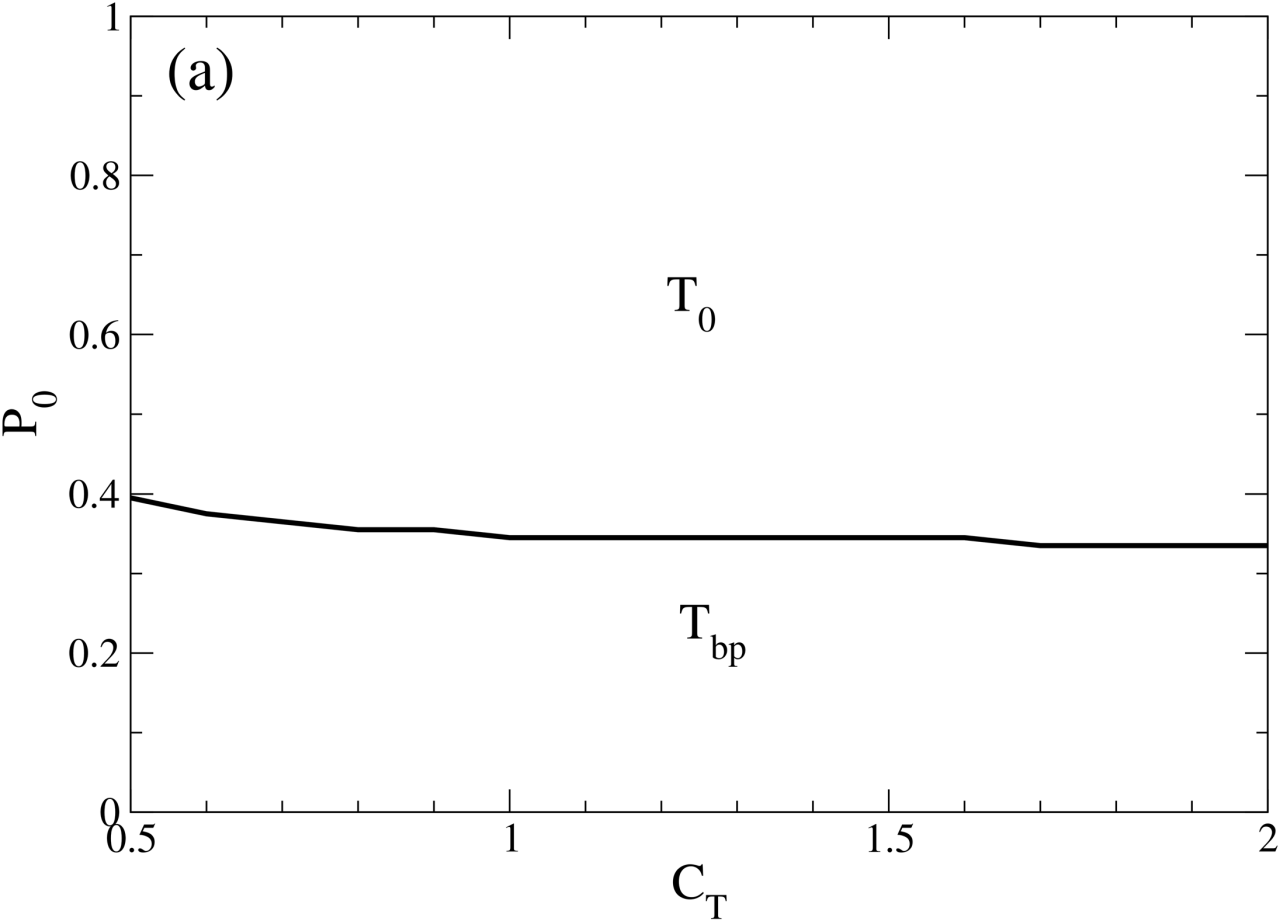
Role of parameter *C*_*T*_. Regions of dominant solution as a function of *P*_0_ and *C*_*T*_ for Δ = 0.01. This plot shows that in the low Δ region and with symmetric distribution of opposing opinions, the system evolves either to bi-polarization or convergence of undecided, depending on the initial fraction of undecided individuals, as have been seen in the Fundamental Phase Diagram (Fig 2).

Increasing the threshold makes the intermediate interval in the persuasion space, which defines agents to be undecided, to be larger. Less undecided are needed initially (at *t* = 0) in order to have more of them at the end. This moves the transition *T*_*bp*_ → *T*_0_ slightly down (see Fig 6).

**Fig 7:**
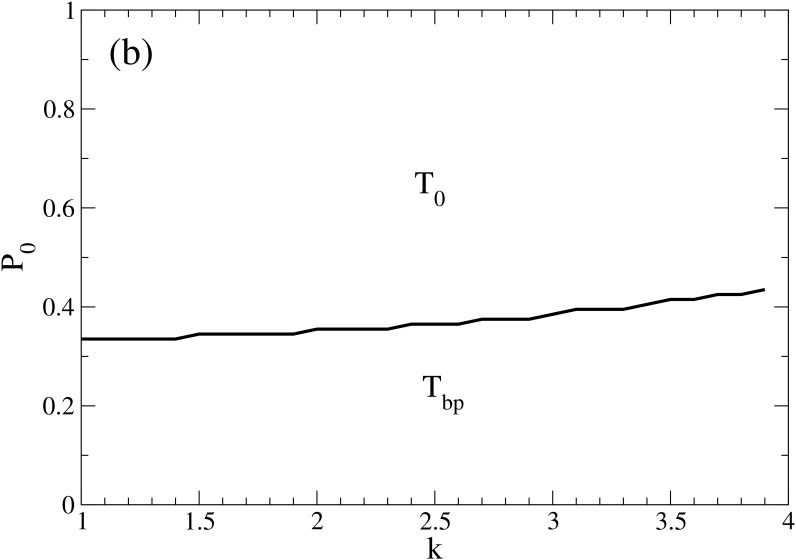
Role of parameter *k*. Regions of dominant solution as a function of *P*_0_ and *k* for Δ = 0.01. This plot shows that in the low Δ region and with symmetric distribution of opposing opinions, the system evolves either to bi-polarization or convergence of undecided, depending on the initial fraction of undecided individuals, as have been seen in the Fundamental Phase Diagram.

Instead, with increasing k, the tendency to adopt a definite opinion (+/ −1) after each interaction grows, and therefore, more undecided individuals are needed in order to achieve a final state where they predominate. Thus, this moves the transition slightly up (see Fig 7). When the asymmetry parameter is changed, the model moves between a pragmatic null “symmetric” model (*k* = 1) and a more extreme asymmetric case (*k* > 2).

### How many undecided are relevant?

In real social situations, the value of *P*_0_ depends on the underlying context, and it may be large or small. Clearly, if we consider examples of voting elections, then assuming very large values of *P*_0_ is less feasible. It is hard to imagine a social system where there is a huge percentage of undecided voters. From the literature and web survey, this number is of orden 10–15%. In fact, voting must be considered carefully because the term “undecided” requires correct precision according to Gordon [40], and Galdi [41].

Instead, social examples of college decision (up to 50% of students enter college as “undecided” [40]) or the choice of major (an estimated 75% of students change their major at least once before graduation [40]) present situations where large values of *P*_0_ are justified.

In the previous section we showed that, when the initial fraction of undecided agents, *P*_0_ is large, the system presents three solutions as a function of Δ: convergence of undecided for small Δ, consensus of one of the two opinions for intermediate values of Δ and bi-polarization for large values of Δ. On the other side, when *P*_0_ is small the equilibrium is only bi-polarization because it is driven mainly by the repulsive force (see Fig 2). Thus, we are interested in the next question: in systems with a relatively small fraction *P*_0_ what should be undertaken in order to obtain the equilibria states observed previously.

**Fig 8:**
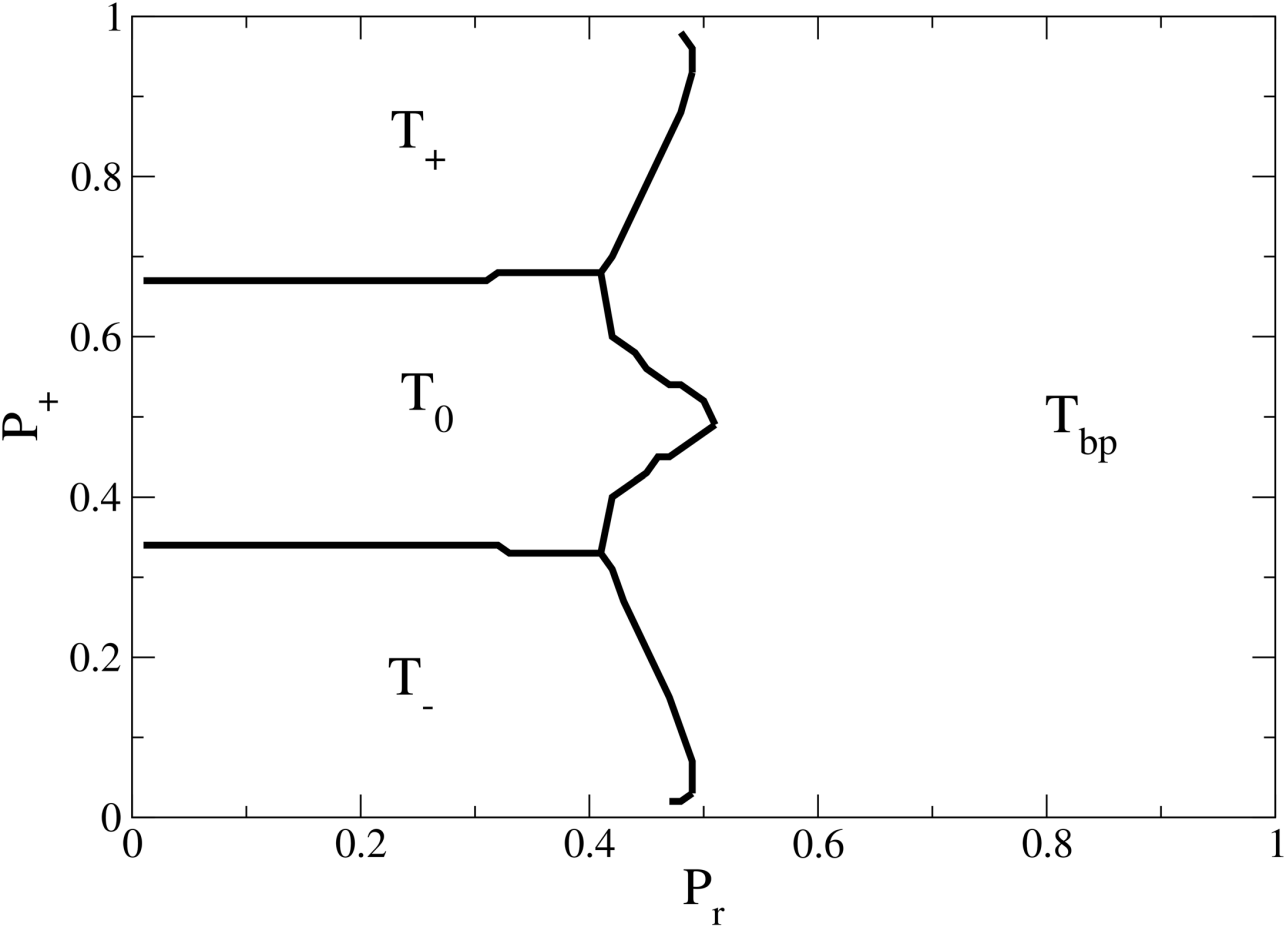
Alternative Phase Diagram. Regions of dominant solution as a function of *P*_+_ (Bias) and *P*_*r*_ (Repulsion probability) for initially low undecided concentration and small Δ (*P*_0_ = 0.10 and Δ = 0.01). If *P*_*r*_ = 1, agents with opposing opinions always repel and the steady state is a polarized situation as we found in the Phase Diagram in Fig 2. When *P*_*r*_ < 0.5, it can happen that agents with opposite opinions get approached enough and the steady state depends on the bias to some opinion. If one of opinions initially prevails (*P*_+_ > 0.68 or *P*_+_ > 0.32), then the population will go to the consensus of this opinion. Otherwise, a convergence of undecided agents is reached.

We propose an alternative scenario for the interaction dynamics. Instead of implemented repulsive effect, allow the individuals with opposing opinions repel with some positive probability *P*_*r*_, in a similar way it was treated in [32]. Also we analyse the effect of breaking the initial symmetry in the distribution of dissimilar opinions. Recall that we call *P*_+_ the fraction of agents with *O* = +1 after the undecided are assigned. The concentration of undecided is fixed. The joint effect of these modifications produce an interesting result. The Phase Diagram for *P*_+_ vs *P*_*r*_ (Repulsion probability) for initially low concentration of undecided agents and a small value of Δ (*P*_0_ = 0.10 and Δ = 0.01) is presented in Fig 8.

When *P*_*r*_ = 1, agents with opposing opinions always repel and the equilibrium is a polarized state as found in the Phase Diagram in Fig 2. When *P*_*r*_ < 0.5, agents sharing opposing opinions may get attracted with probability (1 − *P*_*r*_), and the steady state depends on the bias to any opinion. If any of opinions initially prevails (*P*_+_ > 0.68 or *P*_+_ > 0.32), then the population will reach the consensus to this opinion. Otherwise, a convergence to “all undecided” for is reached.

Both scenarios for the interaction dynamics analysed here are interesting from the social point of view because, in turn, they correspond to the two different sides of the public debate: “Do we learn more from the people with opposing opinions of our own?”. On one side there is a view that we learn much more from people with similar opinions because we do learn more arguments fortifying that belief and take things as facts. On the other side, there is a view that we only learn if we look beyond: in a discussion with people with opposing thoughts we see the different points of view, the exchange of thoughts, etc.

## Discussion

In this manuscript we presented a three-state opinion model based on the cumulative persuasion-threshold dynamics produced by repeated interactions in a social environment. Given that each agent can have either of the two opposing opinions or be in an undecided state, this models applies only for circumstances like a pro-against issue.

We presented the main stationary results of the numerical simulations as a function of the relevant parameters and found that the model is able to reproduce all the expected collective behaviours. In its initial formulation the model shows, as a function of Δ, three different collective states: convergence of undecided, consensus of either of the two opinions and bi-polarization. Multiple stable states are only possible for large values of *P*_0_, the initial fraction of undecided agents. A situation where most of individuals are initially undecided could be presented in low information scenarios about the topic in debate, as for example the discussion about the environmental impact of fracking in US [42], or the choice of major [40].

A paradigmatic scenario of low values of *P*_0_ is, for instance, a two-candidate political election, where typical values of undecided in previous poll give percentages around 10–15%. The initial formulation of the model predicts bi-polarization as the only possible collective state for this range of values of *P*_0_. This is due to the repulsion mechanism assumed in the model by which two individuals with opposing opinions repel from each other. If we relax this condition and let the system start from a non-symmetrical initial condition as was explained in previous section, the model can also show the same three mentioned collective states as a function of *P*_*r*_ and *P*_+_, as is shown in Fig 8.

The presented model has very basic assumptions, as for instance, all the agents are identical (all have the same threshold) and the sensitivity in their persuasions after each interaction is not partner-dependent. Also each agent can interact with everyone and there is no any social network underlying the interaction among them. But even in this simple scenario, the model presents a very rich behaviour, with different collective states appearing in different parameter’s region. Preliminary simulations in this direction (not shown in this work) show that certain types of underlying social networks produce the existence of metastable asymptotic states where a non-negligible fraction of undecided agents still coexists with agents of the two opposite opinions. These kinds of states, which are not possible in the present formulation, are expected to be present, for instance, in a two-candidate political election, where undecided or blank votes is one of the outcomes in the final results. Further analysis in this direction are left for a future work.

Along the results shown in this manuscript, we were able not only do a detailed analysis of the numerical simulations of different features of this model, but also to give a glimpse to a theoretical approach of this model (see appendix). In the corresponding section, we were able to write down the exact master equations for the evolution of the probability of having a given persuasion *C*, *s*_*i*_ (*i* = +, −, 0) and sketch a set of difference equations for this variables. The equations are easily put in context in their continuous version. Then, it can be seen that the master equations are a nonlinear coupled system of first order differential equations of hyperbolic type including nonlocal terms and nonlocal boundary conditions. As long as we know, there are rather few works in solving this kind of equations and the difficulties they present to be solved, as was mentioned in the corresponding section. We let the solution of these equations for future work that is currently under research.

Finally, we would like to mention the potentiality of this model. This formulation is general and covers the social main stream theories of group opinion dynamics. In particular, it is compatible with the persuasive argument theory. The effect of persuasive arguments can be modelled by introducing the set of arguments available to individuals for an interpersonal communication arguments exchange as was done in [31]. The model is also consistent with social decision theory, because from a purely formal point of view, one can assume any mechanism for opinion revision, be it weighted averages of the group initial opinions, or imitation dynamics of the neighbours if the networks of interactions is included, etc. Also, the model may be generalised to self-categorisation theory, similar to Salzarulo [43]. We leave these extensions for future work.

### Appendix: Theoretical Approach

In order to gain a deeper comprehension of the dynamics of this model, we present in this section the deduction of the master equations corresponding to the dynamics of the system.

Briefly, we present here a nonlinear system of nonlocal conservation laws, and let us remark that there are few models of this type even for a single equation. For instance, in the works of Deffuant, Neau, Amblard and Weisbuch, see [44, 45] in opinion dynamic models, among others, where only agents with similar opinions can interact, they obtained nonlocal terms involving a small neighbourhood of a given opinion and they simplify them performing Taylor expansions, recovering local equations of porous media or Fokker-Planck type. Of course, this is possible only in the frame of bounded confidence models, not enabling long range interactions.

Today, nonlinear equations are pervasive in theoretical and applied models, and there exist a growing literature on nonlocal problems. In opinion formation models, we can cite only the work of Aletti, Naldi and Toscani [46], where the mean value of the opinions *m*(*t*) appears in the transport term,

$$\partial_{t}u=\gamma \partial_{x}(\left(1-x^{2}\right)\left(x-m\left(t\right)\right)u),\;\left(x,t\right)\in \left[0,1\right]\times \left(0,\infty \right),$$

here *γ* ∈ [−1, 1], and $m\left(t\right)=\int_{0}^{1}yu\left(y,t\right)dy$, although this term appears only as a mean field effect on the opinion. With a different motivation, inspired in the dislocation dynamic of crystals, Ghorbel and Monneau considered in [47] the following equation:

$$\partial_{t}u=(c\left(x\right)+\int_{R}\varphi \left(u\left(x-y,t\right)\right)dy)\partial_{x}u,\;\left(x,t\right)\in R\times \left(0,\infty \right),$$

and similar equations appeared in continuum mechanics in the theory of deformations and fractures, see for example [48] and the references therein.

It is worth noticing that there are few theoretical results and numerical methods for these problems, which are under active research. Let us mention that the limit as the nonlocal terms shrink to local ones gives an ill-posed problem, i.e., a porous media equation backward in time similar to the ones appearing in the Weisbuch-Deffuant type models. Recall that ill-posed problems lacks the continuity respect to small variations of the initial data, and this explains why the system is difficult to analyze from both the numerical and theoretical point of view. The study of the equations will appear in a separate work, due to the highly technical character of the analysis.

In order to obtain the partial differential equations describing the model, let us look at the system as composed by three populations, according the opinions of the agents. Lets recall than an agent with opinion *O* = +1 has a persuasion *C* ∈ [*C*_*T*_, *C*_*max*_], another with *O* = 0 has *C* ∈ [*C*_*T*_, −*C*_*T*_] and an individual with *O* = −1 has *C* ∈ [−*C*_*max*_, −*C*_*T*_]. We divide the interval corresponding to each opinion in *M* = Δ^−1^ subintervals. Given the evolution of the persuasion according to the interaction-evolution rules detailed in previous sections, the best way to describe the dynamics of the model is in terms of the density of agents with a given persuasion. With this goal we define this density for 1 ≤ *j* ≤ *M* and *t* ≥ 0,

$$s_{+}\left(j,t\right)=\frac{\#\{i\in \left[C_{T}+\left(j-1\right)\Delta ,C_{T}+j\Delta \right)\}}{N},$$

$$s_{0}\left(j,t\right)=\frac{\#\{i\in \left[-C_{T}+\left(j-1\right)\Delta ,-C_{T}+j\Delta \right)\}}{N},$$

$$s_{-}\left(j,t\right)=\frac{\#\{i\in \left[-C_{T}-\left(j-1\right)\Delta ,-C_{T}-j\Delta \right)\}}{N},$$

which represent the fraction of agent of each opinion with persuasion in each interval of length Δ. In this way we can obtain a coupled system of 3*M* difference equations governing the evolution of the density of agents.

We call $S_{i}^{k\leq j}\left(t\right)=\sum_{k=1}^{j}s_{i}\left(j,t\right)$, $S_{i}^{k\geq j}\left(t\right)=\sum_{k=j}^{M}s_{i}\left(j,t\right)$, and recall that *S*_*i*_(*t*) is the fraction of agents with opinion *i*, where *i* ∈ {+, −, 0}.

In the following, we omit the variable *t* in the right hand side of the equations for brevity. After some characteristic time *τ*, depending on the rate of the interactions, we have that the variation on the density of agents is given by the balance between gain and loss terms. For example, when *O* = −1, we have

$$s_{-}\left(j,t+\tau \right)-s_{-}\left(j,t\right)=G_{-}\left(j,t\right)-L_{-}\left(j,t\right)$$

where the term *G*_−_ corresponds to those agents located at *j* − 1 which interact with an agent with opinion *O* = +1 or an agent with opinion *O* = −1 and a stronger persuasion, plus those agents located at *j* + 1 which interact with an agent with neutral opinion *O* = 0 or an agent with opinion *O* = −1 and a weaker persuasion:

$$G_{-}\left(j,t\right)=2s_{-}\left(j-1\right)\left[S_{+}+S_{-}^{k\geq j}\right]+2s_{-}\left(j+1\right)\left[S_{0}+S_{-}^{k\leq j}\right].$$

On the other hand, the loss term corresponds to interactions between an agent located at *j* with another agent in any other location,

$$L_{-}\left(j,t\right)=-2s_{-}\left(j\right)\left[1-s_{-}\left(j\right)\right].$$

So, in this way we obtain the following system of equations:

$$\begin{array}{cc} s_{-}\left(j,t+\tau \right)-s_{-}\left(j,t\right)= & -2s_{-}\left(j\right)\left[1-s_{-}\left(j\right)\right]+2s_{-}\left(j-1\right)\left[S_{+}+S_{-}^{k\geq j}\right]\\ & +2s_{-}\left(j+1\right)\left[S_{0}+S_{-}^{k\leq j}\right], \end{array}$$

$$\begin{array}{cc} s_{+}\left(j,t+\tau \right)-s_{+}\left(j,t\right)= & -2s_{+}\left(j\right)\left[1-s_{+}\left(j\right)\right]+2s_{-}\left(j-1\right)\left[S_{-}+S_{+}^{k\geq j}\right]\\ & +2s_{+}\left(j+1\right)\left[S_{0}+S_{+}^{k\leq j}\right], \end{array}$$

$$\begin{array}{cc} s_{0}\left(j,t+\tau \right)-s_{0}\left(j,t\right)= & -2s_{0}\left(j\right)\left[1-s_{0}\left(j\right)\right]+2s_{0}\left(j-1\right)S_{+}^{k\geq j}+2s_{0}\left(j+1\right)S_{+}^{k\leq j}\\ & +2s_{0}\left(j+k\right)S_{-}+2s_{0}\left(j-k\right)S_{+} \end{array}$$

for 2 < *j* < *M*.

For *j* = 1,2 and *M*, the equations are slightly different, as for instance can be seen for *s*_−_:

$$\begin{array}{cc} s_{-}\left(1,t+\tau \right)-s_{-}\left(1,t\right)= & -2s_{-}\left(1\right)\left[1-s_{-}\left(1\right)\right]+2s_{0}\left(2\right)S_{-}\\ & +2s_{-}\left(2\right)\left[S_{0}+S_{-}^{k\leq 1}\right], \end{array}$$

$$\begin{array}{cc} s_{-}\left(2,t+\tau \right)-s_{-}\left(2,t\right)= & -2s_{-}\left(2\right)\left[1-s_{-}\left(2\right)\right]+2s_{-}\left(1\right)\left[S_{+}+S_{-}^{k\geq 2}\right]\\ & +2s_{-}\left(j+1\right)\left[S_{0}+S_{-}^{k\leq j}\right]+2s_{0}\left(1\right)S_{-}, \end{array}$$

$$\begin{array}{cc} s_{-}\left(M,t+\tau \right)-s_{-}\left(M,t\right)= & -2s_{-}\left(M\right)\left[1-s_{-}\left(M\right)\right]+2s_{-}\left(M-1\right)\left[S_{+}+s_{-}\left(M\right)\right], \end{array}$$

Up to here, we can see that the equations are rather difficult to study, but we can gain more perspective if we move from this discrete version to a continuous model. We can do that by introducing (smooth) functions *u*_*i*_(*x*, *t*), *i* ∈ {0, +, −}, defined for (*x*, *t*) ∈ [0, 1] × [0,∞) such that

$$u_{i}\left(j\Delta ,t\right)=s_{i}\left(j,t\right),$$

and we can approximate the spatial partial derivative as

$$\Delta \partial_{x}u_{i}\left(j\Delta ,t\right)\approx s_{i}\left(j+1,t\right)-s_{i}\left(j,t\right)\approx s_{i}\left(j,t\right)-s_{i}\left(j-1,t\right),$$ (1)

and the temporal partial derivative as

$$\tau \partial_{t}u_{i}\left(j\Delta ,t\right)\approx s_{i}\left(j,t+\tau \right)-s_{i}\left(j,t\right).$$

Also,

$$S_{i}^{k\geq j}\left(t\right)\approx \int_{0}^{j\Delta }u_{i}\left(y,t\right)dy,\;S_{i}^{k\leq j}\left(t\right)\approx \int_{j\Delta }^{1}u_{i}\left(y,t\right)dy,$$

We assume that *τ* = Δ, which corresponds to a time scaling of the rate of interactions.

After some algebra, the continuous version of the master equations reads as

$$\begin{array}{cc} \frac{1}{2}\partial_{t}u_{-}\left(x,t\right)= & \partial_{x}[u_{-}\left(x,t\right)(\int_{0}^{x}\;u_{-}\left(y,t\right)dy-\int_{x}^{1}\;u_{-}\left(y,t\right)dy)]\\ & +\partial_{x}[u_{-}\left(x,t\right)(\int_{0}^{1}\;u_{0}\left(y,t\right)dy-\int_{0}^{1}\;u_{+}\left(y,t\right)dy), \end{array}$$

$$\begin{array}{cc} \frac{1}{2}\partial_{t}u_{+}\left(x,t\right)= & \partial_{x}[u_{+}\left(x,t\right)(\int_{0}^{x}\;u_{+}\left(y,t\right)dy-\int_{x}^{1}\;u_{+}\left(y,t\right)dy)]\\ & +\partial_{x}[u_{+}\left(x,t\right)(\int_{0}^{1}\;u_{0}\left(y,t\right)dy-\int_{0}^{1}\;u_{+}\left(y,t\right)dy), \end{array}$$

$$\begin{array}{cc} \frac{1}{2}\partial_{t}u_{0}\left(x,t\right)= & \partial_{x}[u_{0}\left(x,t\right)(\int_{0}^{x}\;u_{0}\left(y,t\right)dy-\int_{x}^{1}\;u_{0}\left(y,t\right)dy)]\\ & +2\partial_{x}[u_{-}\left(x,t\right)(\int_{0}^{1}\;u_{0}\left(y,t\right)dy-\int_{0}^{1}\;u_{+}\left(y,t\right)dy). \end{array}$$

The boundary conditions for *s*_−_ are given by

$$u_{-}(0,t)=(\frac{\int_{0}^{1}u_{-}(y,t)dy}{\int_{0}^{1}u_{-}(y,t)dy+\int_{0}^{1}u_{+}(y,t)dy})2u_{0}(0,t),$$

$$\begin{array}{c} \partial_{x}u_{-}\left(M,t\right)=0, \end{array}$$

and the ones corresponding to *u*_+_ are similar. The no flux boundary condition at *x* = 1 follows from the assumption that the persuasions are saturated at ±*C*_*max*_. For *u*_0_, there are two non zero Dirichlet boundary conditions similar to the one for *u*_−_(0, *t*), reflecting the incoming agents with opinions *O* = ±1.

In this way we have obtained a nonlinear coupled system of first order differential equations of hyperbolic type including nonlocal terms and nonlocal boundary conditions.

Few remarks are in order:

- We have obtained a system of conservation laws, since the total mass of the solution is conserved, that is, for every *t* ≥ 0,
  $$u_{-}\left(t\right)+u_{+}\left(t\right)+u_{0}\left(t\right)=1.$$
  Some mathematical properties of the solution, like positivity, seems difficult to prove, although the model clearly generates nonnegative solutions.
- The partial differential equation for each opinion has two competing terms: a coalescent one,
  $$\partial_{x}[u_{-}\left(x,t\right)(\int_{0}^{x}u_{-}\left(y,t\right)dy-\int_{x}^{1}u_{-}\left(y,t\right)dy)]$$
  depending on the own distribution *u*_*i*_, which tends to concentrate the agents around the mean value of the opinion *i*; and the other one is a pure transport term,
  $$\partial_{x}[u_{-}\left(x,t\right)(\int_{0}^{1}u_{0}\left(y,t\right)dy-\int_{0}^{1}u_{+}\left(y,t\right)dy),$$
  which drives the population to ±*C*_*max*_, ±*C*_*T*_ depending on the densities of the other two populations, as was shown in previous sections.
- The coalescent terms $\partial_{x}[u_{i}\left(x,t\right)(\int_{0}^{x}\;u_{i}\left(y,t\right)dy-\int_{x}^{1}\;u_{i}\left(y,t\right)dy)]$ changes signs, suggesting the existence of shocks (but perhaps they are smoothed by the transport term). Let us suppose that $\int_{0}^{1}\;u_{i}\left(y,t\right)dy∼C$, and let us call *μ*_*i*_(*t*) the median of the distribution *u*_*i*_, i.e.,
  $$\int_{0}^{\mu_{i}\left(t\right)}u_{i}\left(y,t\right)dy=\frac{C}{2}.$$
  We can rewrite the equation as
  $$\frac{1}{2}\partial_{t}u_{i}=\partial_{x}[u_{i}\left(x,t\right)(2\;\int_{0}^{x}\;u_{i}\left(y,t\right)dy-C)],$$
  and, for *x* ∈ (0, *μ*(*t*)), the characteristic curves travel from left to right, and for *x* ∈ (*μ*(*t*),1) they travel from right to left. Hence, we can expect the formation of a shock curve along the trajectory of *μ*_*i*_(*t*).

We believe that they are out of the scope of this paper and deserve a lengthier discussion.

## References

[1] Schelling TC (1978) Micromotives and Macrobehavior. W. W. Norton Company Ltd.

[2] Granovetter M (1978) Threshold models of collective behavior. American Journal of Sociology 83: 1420–1443 10.1086/226707

[3] Smith PL, Vickers D (1988) The accumulator model of two-choice discrimination. Journal of Mathematical Psychology 32:135–168 10.1016/0022-2496(88)90043-0

[4] Smith PL, Ratcliff R (2004) Psychology and neurobiology of simple decisions. Trends in Neurosciences 27: 161–168 10.1016/j.tins.2004.01.006 15036882

[5] Festinger L (1957) A theory of cognitive dissonance. Evanston, White Plains: Row, Petersen and Company

[6] Homans GC (1951) The human group. New York: Harcourt Press

[7] Heider F (1967) Attitudes and cognitive organization In: Fishbein M, editor, Readings in attitude theory and measurement, new York, London, Sydney: John Wiley and sons, Inc pp. 39–41

[8] Akers RL, Krohn MD, Lanza-Kaduce L, Radosevich M (1979) Social learning and deviant behavior: a specific test of a general theory. American Sociological Review 44: 636–655 10.2307/2094592 389120

[9] Bikhchandani S, Hirshleifer D, Welch I (1992) A theory of fads, fashion, custom and cultural change as informational cascades. Journal of Political Economy 100: 992–1026 10.1086/261849

[10] Friedkin NE (1999) Choice Shift and Group Polarization. American Sociological Review 64:856–875 10.2307/2657407

[11] Eysenck MW (2002) Simply Psychology Madison Avenue, New York, NY: Psychology Press

[12] Baron RS, Hoppe SI, Kao CF, Brunsman B, Linneweh B, Rogers D (1996) Social corroboration and opinion extremity. Journal of Experimental Social Psychology 32: 537–60 10.1006/jesp.1996.0024 8979933

[13] Sanders GS, Baron RS (1977) Is social comparison irrelevant for producing choice shifts? Journal of Experimental Social Psychology 13: 303–14 10.1016/0022-1031(77)90001-4

[14] Vinokur A, Burnstein E (1978) Depolarization of attitudes in groups. Journal of Personality and Social Psychology 36: 872–885 10.1037/0022-3514.36.8.872

[15] Myers DG (1982) Polarizing effects of social interaction In: Brandstatter H and Davis JH and Stocker-Kreichgauer G, editors, Group Decision Making, London: Academic Press pp. 125–161

[16] Turner JC, Hogg MA, Oakes PJ, Reicher SD, Wetherell MS (1987) Rediscovering the social group: A self-categorization theory. Blackwell: Oxford

[17] Oakes PJ, Turner JC, Haslam SA (1991) Perceiving people as group members: The role of fit in the salience of social categorizations. British Journal of Social Psychology 30:125–144 10.1111/j.2044-8309.1991.tb00930.x

[18] Oakes PJ, Haslam SA, Turner JC (1994) Stereotyping and Social Reality. Blackwell: Oxford

[19] Davis JH (1973) Group decision and social interaction: A theory of social decision schemes. Psychological Review 80: 97–125 10.1037/h0020065

[20] Kerr NL (1992) Group decision making at a multialternative task: Extremity, interfaction distance, pluralities, and issue importance. Organizational Behavior and Human Decision Processes 52:64–95 10.1016/0749-5978(92)90046-A

[21] Hastie R, Kameda T (2005) The Robust Beauty of Majority Rules in Group Decisions. Psychological Review 112: 494–508 10.1037/0033-295X.112.2.494 15783295

[22] Castellano C, Fortunato S, Loreto V (2009) Statistical physics of social dynamics. Rev Mod Phys 81:591–646 10.1103/RevModPhys.81.591

[23] Latané B (1981) The psychology of social impact. American Psychologist 36: 343–356 10.1037/0003-066X.36.4.343

[24] Weisbuch G, Deffuant G, Amblard F, Nadal J-P (2002) Meet, discuss and segregate! Complexity 7:55–63 10.1002/cplx.10031

[25] Galam S (2005) Heterogeneous beliefs, segregation, and extremism in the making of public opinions. Physical Review E 71:046123 10.1103/PhysRevE.71.046123 15903742

[26] Mäs M, Flache A, Helbing D (2010) Individualization as driving fource of clustering phenomena in humans. PloS Comput Biol 6: e1000959 10.1371/journal.pcbi.1000959 20975937 PMC2958804

[27] Sznajd-Weron K, Sznajd J (2000) Opinion evolution in closed community. Int. J. Mod. Phys. C 11:1157–1165 10.1142/S0129183100000936

[28] Abelson RP (1964) Mathematical models of the distribution of attitudes under controversy In: Fredericksen N. and Gullicksen H., editors, Contributions to mathematical psychology, Holt, Rinehart & Winston

[29] DeGroot MH (1974) Reaching a consensus. Journal of the American Statistical Association 69: 118–121 10.1080/01621459.1974.10480137

[30] Berger RL (1981) A Necessary and Sufficient Condition for Reaching a Consensus Using DeGroot’s Method. Journal of the American Statistical Association 76: 415–418 10.1080/01621459.1981.10477662

[31] Mäs M, Flache A (2013) Differentiation without distancing. Explaining bi-polarization of opinions without negative influence. PloS ONE 8: e74516 10.1371/journal.pone.0074516 24312164 PMC3842239

[32] La Rocca C E, Braunstein LA, Vazquez F (2014) The influence of persuasion in opinion formation and polarization. EPL (Europhysics Letters) 106: 40004 10.1209/0295-5075/106/40004

[33] Souza SR, Gonçalves S (2012) Dynamical model for competing opinions. Physical Review E 85: 056103 10.1103/PhysRevE.85.056103 23004817

[34] de la Lama MS, Szendro IG, Iglesias JR, Wio HS (2006) Van Kampen’s expansion approach in an opinion formation model. The European Physical Journal B—Condensed Matter and Complex Systems 51: 435–442 10.1140/epjb/e2006-00232-8

[35] Couzin ID, Ioannou CC, Demirel G, Gross T, Torney CJ, Hartnett A, Conradt L, Levin SA, Leonard N E (2011) Uninformed Individuals Promote Democratic Consensus in Animal Groups. Science 334: 1578–1580 10.1126/science.1210280 22174256

[36] Sobkowicz P (2012) Discrete model of opinion changes using knowledge and emotions as control variables. PloS ONE 7: e44489 10.1371/journal.pone.0044489 22984516 PMC3439419

[37] Vázquez F, Redner S (2004) Ultimate fate of constrained voters. Journal of Physics A: Mathematical and General 37:8479 10.1088/0305-4470/37/35/006

[38] Wood W (2000) Attitude change: persuasion and social influence. Annual Review of Psychology 51: 539–570 10.1146/annurev.psych.51.1.539 10751980

[39] Sidoti L (2008) Undecided voters not satisfied with both candidates. Associated Press

[40] Gordon V (2007) The Undecided College Student: An Academic and Career Advising Challenge. Charles C Thomas Publisher, Limited

[41] Galdi S, Arcuri L, Gawronski B (2008) Automatic Mental Associations Predict Future Choices of Undecided Decision-Makers. Science 321: 1100–1102 10.1126/science.1160769 18719288

[42] Boudet H, Clarke C, Bugden D, Maibach E, Roser-Renouf C, Leiserowitz A (2014) “Fracking” controversy and communication: Using national survey data to understand public perceptions of hydraulic fracturing. JEPO Energy Policy 65: 57–67 10.1016/j.enpol.2013.10.017

[43] Salzarulo L (2006) A Continuous Opinion Dynamics Model Based on the Principle of Meta-Contrast. Journal of Artificial Societies and Social Simulation 9: 13

[44] Deffuant G, Neau D, Amblard F, Weisbuch G (2000) Mixing beliefs among interacting agents. Adv Complex Syst 3: 87–98 10.1142/S0219525900000078

[45] Weisbuch G, Deffuant G, Amblard F (2005) Persuasion dynamics. Physica A: Statistical Mechanics and its Applications 353: 555–575 10.1016/j.physa.2005.01.054

[46] Aletti G, Naldi G, Toscani G (2007) First-Order Continuous Models of Opinion Formation. SIAM Journal of Applied Mathematics 67: 837–853 10.1137/060658679

[47] Ghorbel A, Monneau R (2010) Well-posedness and numerical analysis of a one-dimensional non-local transport equation modelling dislocations dynamics. Math Comput 79: 1535–1564 10.1090/S0025-5718-10-02326-4

[48] Du Q, Kamm JR, Lehoucq RB, Parks ML (2012) A New Approach for a Nonlocal, Nonlinear Conservation Law. SIAM Journal of Applied Mathematics 72: 464–487 10.1137/110833233

